# Retraction: Eupalinolide J suppresses the growth of triple-negative breast cancer cells via targeting STAT3 signaling pathway

**DOI:** 10.3389/fphar.2025.1639679

**Published:** 2025-06-11

**Authors:** Frontiers Editorial Office

The journal retracts the 19 September 2019 article cited above.

Following publication, concerns were raised regarding the integrity of the images in the published figures. The authors failed to provide a satisfactory explanation during the investigation, which was conducted in accordance with Frontiers’ policies.

This retraction was approved by the Chief Editors of Frontiers in Pharmacology and the Chief Executive Editor of Frontiers. The authors received a communication regarding the retraction and had a chance to respond. This communication has been recorded by the publisher.

Frontiers would like to thank the users on PubPeer for bringing the published article to our attention.

